# 

**Published:** 1854-08

**Authors:** 


Our next number will contain an account of the various meteorological conditions of the season, which we think will be found somewhat remarkable and interesting,

H.

*Starling Medical College.*—There has been a reorganization of this very efficient school. Our friends, Prof's Lee and Paddock, give up their respective professorships. J. W. Hamilton, M. D., taking the chair of Materia Medica and Jurisprudence, and John Dawson, M. D., the Anatomical chair.

Prof. Moore, of Rochester, has been appointed to the chair of Surgery, and will soon remove to Columbus, to take up his permanent residence. Dr. Moore is a highly educated and skillful surgeon, has passed through a rich experience, and has all the qualities as a man and a lecturer to make himself deservedly popular in the west. H.

---

*Buffalo Medical College.*—The announcement for the next term is now out, and all students or practitioners, who may not receive a copy, may obtain one by addressing a note to any of the resident faculty.

The preliminary term will commence on Monday the 16th of October, and be devoted principally to practical anatomy, to clinical teaching at the Buffalo Hospital of the Sisters of Charity, and to lectures on some special branches of anatomy.

The regular term begins on the first of November, and will continue sixteen weeks. It will not fall behind the usual reputation of the school in interest and profit.

Some erroneous ideas having gone abroad relative to the expenses of living in Buffalo, we may state that they are not larger than in any other large town, while the advantages of clinical teaching are such as no other school affords, if we estimate the number and variety of forms of disease brought before the class, and the convenience and cheapness of the Hospital accommodations. Dissecting material will be abundantly provided at a much cheaper rate than heretofore. H.

---

*The wet sheet in Cholera.*—Our friend Prof. Rochester, some time since, suggested to us that packing in the wet sheet might be a desirable remedy in cholera, and that the blood might thus receive again some portion of the water it had lost. Since then we have seen something of its effects. In one case, where it was applied to a man in collapse and apparently dying, it was followed by reaction and recovery. In no other case could we attribute a cure to it, but we found that in all to whom it was applied it removed the blueness of the skin, and encouraged reaction. In all cases it entirely relieved the cramps, and was a most grateful application to the sufferer. We shall have more to say about it hereafter.

